# Favorable Changes in Lipid Profile: The Effects of Fasting after Ramadan

**DOI:** 10.1371/journal.pone.0047615

**Published:** 2012-10-24

**Authors:** Abdullah Shehab, Abdishakur Abdulle, Awad El Issa, Jassim Al Suwaidi, Nico Nagelkerke

**Affiliations:** 1 Department of Internal Medicine, Faculty of Medicine and Health Sciences, UAE University, Al-Ain, United Arab Emirates; 2 Department of Cardiology and Cardiovascular Surgery, Hamad General Hospital, Doha, Qatar; 3 Department of Community Medicine, Faculty of Medicine and Health Sciences, UAE University, Al-Ain, United Arab Emirates; University of South Florida College of Medicine, United States of America

## Abstract

**Aims:**

We assessed the effect of fasting during Ramadan on blood pressure (BP), body weight, plasma lipid, and lipoprotein variables among healthy normal individuals.

**Methods:**

102 (68% male) multi-ethnic volunteers; mean age ± SD (38.7±10.5 years) were randomly recruited in Al-Ain, United Arab Emirates (UAE), to be investigated before Ramadan, one day after the end of Ramadan, and four weeks after Ramadan. Anthropometric, demographic, fasting plasma total cholesterol (TC), triglyceride (TG), and high density lipoprotein–cholesterol (HDL-C) were measured by standard methods, and Low density lipoprotein-cholesterol (LDL-C) was calculated using Friedewald’s formula.

**Results:**

65 subjects completed the study. We found significant and beneficial changes in systolic blood pressure (SBP), body weight, waist circumference (WC), TG, HDL-C and LDL-C, at the end of Ramadan, but not in TC. Further, there was a progressive and significant increase and decrease in HDL-C and LDL-C levels, respectively, four weeks after Ramadan.

**Conclusions:**

We observed significant improvements in HDL-C, and LDL-C levels even after four weeks post Ramadan. Ramadan-like fasting may be considered for more effective lipid and lipoprotein control.

## Introduction

Fasting in the month of Ramadan, the ninth lunar month of the Islamic calendar, is a religious obligation for all adult Muslims, ordained, in part, to teach self-restraint and body purification [1]. Fasting individuals are required to abstain from eating, drinking, and any form of intimate behaviour for approximately 12 hours (from dawn to sunset) depending on the geographical location [2].

Undoubtedly, such religious obligations may provide an opportunity to a reduced frequency and quantity of food-intake, both of which may lead to plausible health benefits including weight loss, and favourable metabolic changes [3], [4]. However, such weight loss during Ramadan may not be sustained [5]. It is noteworthy, that whilst reduced energy intake may lead to weight loss [6], skipping meals on the other hand may induce weight gain, presumably, due to over-compensatory eating habits [7].

Fasting in Ramadan has been shown to have some effects on the circulating levels of several biochemical markers known to be associated with vascular and metabolic disorders including lipid profile [8], [9], [10]. Farshidfar *et al* reported a significant increase in high density lipoprotein - cholesterol (HDL-C) and a decrease in low density lipoprotein – cholesterol (LDL-C) at day 28 of Ramadan [11]. Also, studies among patients with type II diabetes mellitus reported decreased total cholesterol (TC), triglyceride (TG), and LDL-C as well as increased HDL-C levels after fasting in Ramadan [12].

The changes in lipid profile, however, may vary depending on the quality and quantity of food intake, and physical activity [13]. Moreover, there are regional disparities in dietary habits depending on cultural rituals, often practices during Ramadan, among Muslim societies. Consequently such disparities may affect various components of metabolic importance [14]. Other lifestyle changes, most notably, the more frequent and voluntary prayers performed during Ramadan which is comparable to moderate exercise [15], may lead to a healthier outcome.

To further explore the generalizability of the beneficial effects of Ramadan, we investigated the effects of Ramadan on body weight, lipid profile, blood pressure, and whether or not changes in these parameters, if any, could be maintained four weeks after Ramadan among healthy adults in a Gulf Arab population.

### Subjects and Methods

The Al-Ain Medical District Human Research Ethics Committee approved all study protocols. We invited staff from our University in Al-Ain (UAE) to participate. Of these 102 were enrolled and signed informed consent, but only 65 completed the follow-up procedures. Participants were all Muslim adults of multi-ethnic origin. Trained staff interviewed the subjects and took measurements during the first day of Ramadan (pre-Ramadan), the last day of Ramadan, and four weeks later (post-Ramadan). At each visit, subjects gave a 4 ml blood sample collected EDTA tubes after an overnight fast of at least 12 hours. Samples were separated at 3000 rpm (for 10 minutes @ 4°C). Fresh aliquots were used to measure lipids and lipoproteins.

Height and weight were measured by a calibrated electronic scale and a stadiometer manufactured by Seca Germany (Model 769; Seca, Hamburg, Germany). Prior to measurements, subjects were asked to wear light clothing, but no shoes. Height was taken at standing position with heads, backs and buttocks vertically aligned to the height gauge and the result was rounded to the nearest 0.5 cm, and weight was recorded and rounded to the nearest 0.5 kg. A standard tape was used to measure waist circumference (WC) at a point right above the iliac crest on the midaxillary line at minimal respiration and was rounded to the nearest 1.0 cm. Three measurements of height, weight, and WC were recorded for each subject and averaged for analysis. Body mass index (BMI), the ratio of weight in kilograms to height in square meters [Weight (kg)]/[height (m)]^2^, was calculated to the nearest decimal place.

Blood pressure (BP) was measured using validated automatic monitors (model M6, Omron healthcare, Kyoto, Japan) [16]. Prior to taking BP readings, all subjects rested for five minutes in an air-conditioned environment. Measurements were taken three times, on the right arm using an appropriate cuff size with short intervals between readings, and the average of the last two readings was used for analysis [17].

Lipids profile were measured by a validated point of care portable machine “CardioChek” (Polymer Technology Systems Inc, Indianapolis, IN, USA). LDL-C was estimated using Friedewald’s equation [18].

### Statistics

Statistical analyses were performed using the Statistical Package for Social Sciences (SPSS) version 19.0 for Windows. Standard univariate and multivariate methods, such as regression analysis were used. Student’s t-test and mixed model ANOVA were used to compare results. A p-value <0.05 was considered statistically significant.

## Results

**Table 1** shows anthropometric, blood pressure, and biochemical parameters, by sex, and time (pre- Ramadan, Ramadan, and post Ramadan). Of the 65 participants 70.8% were male.

**Figure 1:**
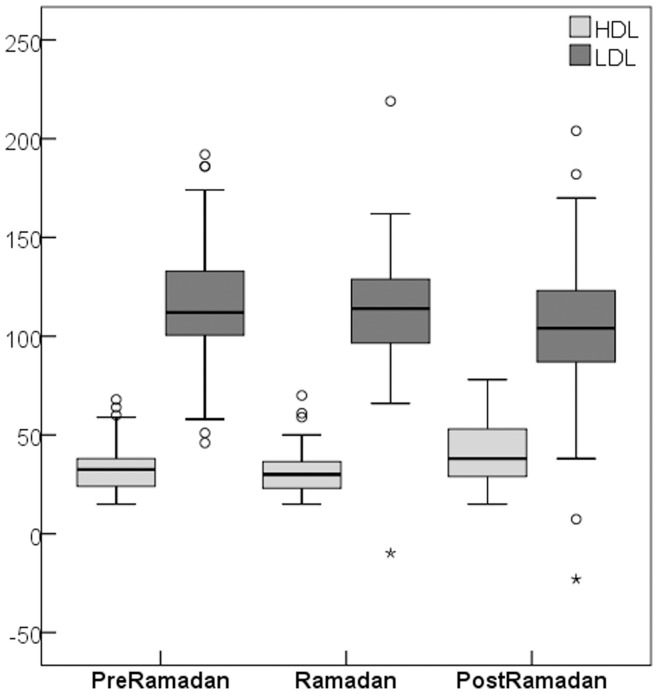
Box plot chart showing the progressive changes in HDL-C (light gray boxes) and LDL-C (dark gray boxes) over the time points.

Overall, SBP, Weight, WC, BMI were significantly lower during Ramadan than either before or after. Interestingly, progressive changes in the levels of lipoproteins were most favorable (higher HDL-C, lower LDL-C) 4 weeks after Ramadan (figure 1). Females tended to have slightly more favorable cardiovascular risk profile than males.

**Table 1:** Comparison of changes in blood pressure, Wt, WC, BMI, lipids, and lipoproteins levels before Ramadan (pre-Ramadan), Ramadan, and four weeks after Ramadan (post-Ramadan); using MIXED ANOVA. (n = 65).

	Gender							
	Male (n = 42)			Female (n = 18)			P-Value	P-Value
	Pre-Ramadan	Ramadan	Post-Ramadan	Pre-Ramadan	Ramadan	Post-Ramadan	For sex	For time
Variables	Mean (SD)	Mean (SD)	Mean (SD)	Mean (SD)	Mean (SD)	Mean (SD)		
Age (years)	43.2 (9.4)	–	–	35.3 (9.1)	–	–	–	–
Height (cm)	171.5 (7.3)	–	–	158.4 (6.2)	–	–	–	–
SBP (mmHg)	124.1 (14.6)	120.8 (13.8)	124.8 (16.3)	117.6 (12.3)	113.5 (11.2)	113.6 (8.9)	**0.017**	**0.02**
DBP (mmHg)	84.7 (10.2)	82.8 (10.0)	84.5 (12.4)	80.2 (9.7)	81.2 (7.9)	77.8 (6.9)	0.091	0.536
Pulse (p/s)	69.6 (10.1)	67.9 (9.6)	71.8 (9.8)	73.2 (8.3)	74.5 (7.2)	72.5 (6.5)	0.116	0.226
Weight (kg)	82.9 (14.6)	81.8 (14.6)	82.4 (15.1)	68.5 (15.1)	67.9 (15.3)	68.3 (14.9)	**0.001**	**0.003**
WC (cm)	96.9 (10.9)	94.5 (11.1)	96.2 (11.0)	79.6 (14.9)	77.2 (15.1)	76.7 (14.5)	**0.001**	**0.001**
BMI (kg/m^2^)	28.1 (4.4)	27.8 (4.4)	28.0 (4.6)	27.2 (5.5)	27.0 (5.7)	27.2 (5.5)	0.503	**0.003**
TC (mmol/L)	4.7 (0.7)	4.4 (0.6)	4.3 (0.7)	4.1 (0.6)	4.0 (0.4)	4.4 (1.1)	0.069	0.062
TG (mmol/L)	1.2 (1.0)	1.1 (1.0)	1.1 (0.9)	0.7 (0.5)	1.2 (1.2)	1.6 (1.4)	0.946	0.254
HDL-C (mmol/L)	0.8 (0.2)	0.8 (0.3)	0.9 (0.3)	0.9 (0.4)	0.9 (0.4)	1.4 (0.3)	**0.001**	**0.001**
LDL-C (mmol/L)	3.3 (0.8)	3.2 (0.7)	2.9 (0.7)	2.8 (0.6)	2.5 (0.6)	2.3 (1.3)	**0.001**	**0.001**
HDL/LDL ratio	0.3 (0.1)	0.2 (0.1)	0.3 (0.1)	0.3 (0.1)	0.4 (0.2)	0.8 (2.1)		

SD; Standard Deviation, SBP; systolic blood pressure, DBP; diastolic blood pressure, Wt; weight, WC; waist circumference, TC; total cholesterol, TG; triglyceride, HDL-C; high density lipoprotein-C, LDL; low density lipoprotein, P-values are for comparison among time points.

## Discussion

Unhealthy lifestyle characterized by excessive consumption of diets high in saturated fatty acids and refined carbohydrates as well as lack of regular exercise is an underlying cause of metabolic disorders including obesity, dyslipidaemia, diabetes, and hypertension [1]. In contrast, maintaining a balanced diet of moderate energy intake, and a regular exercise not only decreases body fat and increases muscle mass, but also it plays an important role in the prevention and management of obesity and sequelae [12]. In this context, the obligatory fasting in Islam may provide an interesting opportunity to reduce food intake and increase physical activity. Whilst such possibilities of reducing food intake may vary from person to person, the extensive extra congregational prayers seem to be universally adopted.

These prayers include; ‘*Tarawih*’ that is performed approximately 1–2 hours after sunset (depending on time zone); unlimited number of non-obligatory ‘*Nafl*’ prayers; and ‘*Tahajud*’ that is performed after midnight at least in the last 10 days, may, arguably, constitute adequate level of physical activity equivalent to moderate physical activity.

Our study subjects were healthy normal individuals who strictly fasted throughout the month of Ramadan. In this study, we report several important findings.

First, a slight weight loss of less than 1 Kg was observed in all subjects at the end of Ramadan compared to the baseline data, but most of our subjects re-gained weight four weeks post-Ramadan. Such pattern of weight loss and re-gain among fasting individuals has been shown previously [5], [15]. The relatively small weight loss found in our study may be explained, in part, by a mild dehydration due to fluid restriction during Ramadan [19]. In spite, the effect of dehydration may have been modest as our data was collected in winter. Changes in the circadian rhythm of eating, though presumed to differ from involuntary food deprivation, and its possible effects of weight loss during Ramadan, may have been masked by nocturnal over-consumption of foods high in carbohydrate and fat contents.

Undoubtedly, weight reduction, even in less than 5% [20], may result in clinically important health improvements, with fewer propensities for Type 2 diabetes, hypertension, heart disease, and endothelial dysfunction [21], [22].

Undoubtedly, the essence of fasting in Ramadan is not only to lose weight as much as it is spiritual. Thus, the benefits of Ramadan may well exceed those that accrue from dieting. Hence, Ramadan does seem to be a good time to implement educational awareness programs about the benefits of healthier lifestyles.

We observed significant reductions in blood pressure levels at the end of Ramadan, perhaps, due to catecholamine inhibition during hunger [23]. Moreover, both TC, and TG were slightly lower at the end of Ramadan, but not four weeks after Ramadan, as compared to baseline. This is in contrast to results from Morocco [24], where a significant reduction in TC and TG levels was reported even four weeks after Ramadan. This was associated with increased consumption, during Ramadan, of monounsaturated and polyunsaturated fatty acids as well as decreased consumption of saturated fatty acids [18]. Another study from Kuwait showed no significant changes in TC and TG levels [25]. Perhaps, such discrepancies in lipid reduction could be explained by the significant differences between the classical Mediterranean dietary habits in Morocco, and the affluent diet, high in saturated fat, in the Arabian Gulf States. However, as we did not collect details on the actual food items consumed we are unable to explore this further. In contrast to TC and TG, changes in HDL-C and LDL-C levels were more favorable even after four weeks of cessation of fasting. Similar changes in HDL-C and LDL-C levels were previously reported in other studies [25], [26], [27], [28], [29], and may suggest that observing Ramadan Fast may constitute a non-pharmacological method to ameliorate lipid disorders. However, whether similar patterns of improvement, seen in these healthy volunteers, would be possible among subjects with lipid disorders is unclear.

Despite the difficulties associated with disentangling the effects of the multiple changes during Ramadan (different food, more prayers, different eating times, dehydration etc), our results highlight potentially relevant health benefits of Ramadan.
